# Stratification of Pro-Atherogenic Phenotypes in Prediabetes Using Machine Learning

**DOI:** 10.3390/biomedicines14030651

**Published:** 2026-03-13

**Authors:** Liana Signorini, Waldemar Volanski, Ademir Luiz do Prado, Glaucio Valdameri, Mauren Isfer Anghebem, Vivian Rotuno Moure, Marcel Henrique Marcondes Sari, Geraldo Picheth, Fabiane Gomes de Moraes Rego

**Affiliations:** 1 Graduate Program in Pharmaceutical Sciences, Laboratory of the Research Group on Metabolic Diseases, Federal University of Parana, Curitiba 80210-170, Brazil; lianasig@ufpr.br (L.S.); ademirlp@ufpr.br (A.L.d.P.); mauren.isfer@ufpr.br (M.I.A.);; 2 Laboratory Division of the Curitiba City Hall, Curitiba 81050-290, Brazil; 3 Department of Computer Science, Federal Institute of Parana, Colombo 83403-515, Brazil; 4 Graduate Program in Pharmaceutical Sciences, Laboratory of Cancer Drug Resistance, Federal University of Parana, Curitiba 80210-170, Brazil; gvaldameri@ufpr.br (G.V.); vivian.moure@ufpr.br (V.R.M.); 5 School of Medicine and Life Sciences, Pontifical Catholic University of Parana, Curitiba 80215-901, Brazil

**Keywords:** binomial logistic regression, cluster, intermediate hyperglycemia, k-means, knowledge discovery database, lipid biomarkers, artificial intelligence, ROC curve

## Abstract

**Background/Objectives:** Prediabetes is a metabolic condition involving various phenotypes of glucose metabolism. Prediabetes increases the risk of heart disease, among other conditions. Hence, we employed machine learning tools to characterize phenotypes associated with cardiovascular disease using routine laboratory biomarkers. **Methods:** We processed laboratory records of over 1,000,000 de-identified individuals, resulting in a sample of 3024 individuals classified as prediabetic (fasting blood glucose 100–125 mg/dL combined with HbA1c 5.7–6.4%). Lipid profile parameters (total cholesterol [TC], HDL-C, LDL-C, and triglycerides) and associated indices (atherogenic index of plasma, Log_10_(TG/HDL-C), triglyceride–glucose index [TyG], TC/HDL-C, and LDL-C/HDL-C, among others) were analyzed using the k-means algorithm. Two groups emerged based on biomarker concentrations, a pro-atherogenic cluster (P-AC; n = 1113) and a less-atherogenic cluster (L-AC; n = 1911) for cardiovascular disease. **Results:** We assessed the performance of biomarkers in the P-AC and L-AC clusters using a receiver operating characteristic curve. Triglycerides (area under the curve [AUC] 0.977), AIP (AUC 0.978), and triglyceride–glucose index (AUC 0.974) showed sensitivity and specificity >90%. The TC/HDL-C (AUC 0.903) and LDL-C/HDL-C (AUC 0.865) indices also performed well, with sensitivity and specificity of 80%. Binomial logistic regression applied to the groups generated by k-means using the biomarkers AIP and LDL-C/HDL-C showed an AUC of 0.984 and accuracy above 93%. **Conclusions:** The k-means algorithm enabled the identification of a P-AC for cardiovascular disease among prediabetics using cost-effective laboratory biomarkers that are widely accessible in laboratories. Individuals classified as P-AC may benefit from differentiated treatment to minimize this factor.

## 1. Introduction

Prediabetes, also known as intermediate hyperglycemia, is characterized by an imbalance in carbohydrate metabolism, with glycemic biomarker concentrations that fall between normal blood sugar levels and those defined under diabetes criteria [1]. The progression of prediabetes to type 2 diabetes (T2D) is often silent, posing a significant public health challenge. Notably, 30% to 50% of individuals with prediabetes remain undiagnosed [2]. Approximately 25% of people with prediabetes will progress to T2D during an asymptomatic period, known as the presymptomatic phase, which can last for 3–12 years [1,3]. This progression results from a combination of decreased tissue sensitivity to insulin (insulin resistance) and a progressive decline in pancreatic insulin secretion [4,5,6].

**Figure 1:**
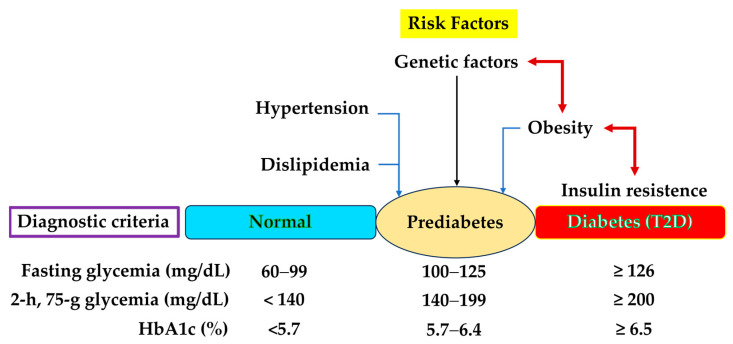
Risk factors and diagnostic criteria for diabetes and prediabetes. According to the American Diabetes Association [1], prediabetes is defined as blood glucose above normal levels but not yet high enough to be diagnosed as T2D, and the diagnostic criteria include fasting glucose measured in the morning after 8 h of fasting, 100–125 mg/dL (5.6–6.9 mmol/L); 2 h glucose after a 75 g glucose dose during the oral glucose tolerance test with 75 g glucose dose, 140–199 mg/dL (7.8–11.0 mmol/L); and Hb_A1c_, the glycated hemoglobin fraction A1c, 5.7–6.4% (39–46 mmol/mol). Visceral obesity affects insulin sensitivity, increases the secretion of inflammatory cytokines, and promotes hypertension and atherogenesis, leading to both micro- and macrovascular complications [4].

Figure 1 illustrates the main characteristics of prediabetes, highlighting the classification criteria proposed by the American Diabetes Association (2025) and the Brazilian Diabetes Society [1,7].

The prevalence of prediabetes is significant, with variations according to a country’s income, ethnicity, and the diagnostic criteria used, showing a global prevalence of 12% using the fasting blood glucose criteria and 9.2% with the 2 h oral glucose tolerance test criteria [2]. The number of individuals with prediabetes is increasing, parallel to the global T2D epidemic.

From a clinical standpoint, individuals with prediabetes often present additional risk factors such as dyslipidemia, hypertension, overweight, obesity, a sedentary lifestyle, smoking, age >35 years, a history of gestational diabetes, and genetic predispositions to several metabolic and systemic conditions [3,4,8,9]. The presence of metabolic syndrome in prediabetes is common, with significant atherogenic changes in the lipid profile, including increased triglycerides (TG), total cholesterol (TC), LDL-cholesterol (LDL-C), VLDL-cholesterol (VLDL-C), and the TG/HDL-C ratio, alongside decreased HDL-cholesterol (HDL-C) [4,5,9]. In addition, a 10 mg/dL increase in serum TGs is associated with a 4% increased risk for T2D [5].

The prediabetic group is heterogeneous, encompassing diverse risk phenotypes for T2D and other pathologies [10]. It is advantageous to identify individuals with prediabetes who are at higher risk for other complications, especially for public health services. Lifestyle interventions can reduce the progression from prediabetes to T2D by 58% over three years, highlighting the need for early prevention strategies and specific public policies to reduce the burden on health systems [5].

The economic and healthcare impact of prediabetes is significant, resulting from the increased risk of cardiovascular complications associated with moderate hyperglycemia, dyslipidemia, oxidative stress, the formation of advanced glycation end products, endothelial dysfunction, and a prothrombotic state, which precedes conversion to overt T2D [9].

Cardiovascular diseases (CVDs) are the leading causes of death and disability worldwide [11]. In the pre-diabetes stage, traditional risk factors for CVD, such as dyslipidemia, obesity, and hypertension, are prevalent [12]. Studies suggest that the association with CVD is not present in all prediabetics but is primarily evident in those with hypertension, indicating that this group may represent a specific phenotype [13].

Defining phenotyping strategies for individuals with prediabetes is essential for implementing more effective intervention measures for those at high risk of developing T2D and associated complications such as CVDs, as response to therapeutic strategies differs between these pathological processes [4].

Artificial intelligence (AI) models and algorithms have been successfully employed to separate or classify heterogeneous groups such as prediabetes [14]. For instance, Matboli et al. (2025) used machine learning classifiers integrated with laboratory and molecular biomarkers to classify different stages of diabetes [15]. Such methods have demonstrated efficiency and cost-effectiveness in studies assessing the risk of developing diabetes in large databases [16], in addition to showing favorable responses in low- and middle-income countries due to reduced processing costs [17].

Given this context, we aimed to apply AI models associated with routine laboratory biomarkers to a selected group of prediabetics to stratify those with pro-atherogenic characteristics for CVD. This study highlighted the use of the k-means algorithm and binomial logistic regression statistics, both machine learning models, a subset of AI, widely used as exploratory methods for cluster discrimination. The proposed laboratory biomarkers are low-cost and accessible through the public health system.

## 2. Materials and Methods

### 2.1. Sample

The database consists of de-identified records obtained from the Laboratory Information System of the Municipal Laboratory of Curitiba (Curitiba, Paraná State, Brazil), a tertiary laboratory serving the municipal Public Health Service. The study was approved by the Research Ethics Committee (CAAE no. 68027317.7.0000.0102). The records of anthropometric data (age and sex) associated with TC, HDL-C, TGs, fasting glycemia (GLY), and HbA1c levels were selected over a one-year period. The records used did not include information on medication use, family history, or the presence of other comorbidities.

The inclusion criteria included records of patients who presented concomitant GLY levels of 100–125 mg/dL and HbA1c of 5.7–6.4%, as well as lipid profile parameters. The cut-off values for biomarkers to characterize prediabetes are recommended in a widely used global guideline [18], as shown in Figure 1. As for the exclusion criteria, records of patients aged <18 years, duplicate data from the same patient, pregnancy, and incomplete data among the analyzed parameters were excluded.

### 2.2. Laboratory Quantifications

**Figure 2:**
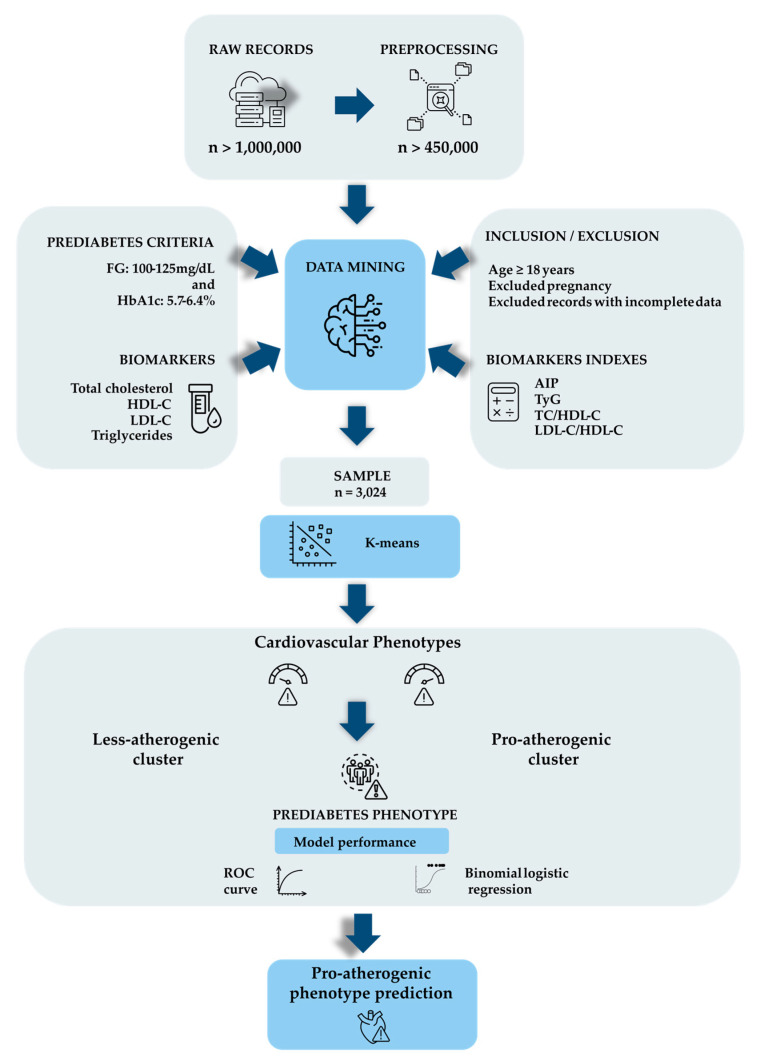
Data flow diagram. Over 1,000,000 de-identified records obtained from laboratory databases were processed to remove homonyms, redundancies, and incomplete data (n > 450,000). These data were then applied to pre-diabetic criteria, cardiovascular risk biomarkers, and inclusion and exclusion criteria, resulting in a sample (n = 3024). The k-means algorithm was used to classify the data into two groups: pro-atherogenic cluster and less-atherogenic cluster for CVD. These groups were evaluated using receiver operating characteristic (ROC) curves and binomial logistic regression (BLR) to predict pro-atherogenic phenotypes in prediabetes.

Samples were collected after 12 h of fasting. Hb_A1c_ was quantified in whole blood (EDTA K2, Vacutainer, Becton Dickinson, Franklin Lakes, NJ, USA) using high-performance liquid chromatography with an ion-exchange column (Variant II Turbo, BioRad Laboratories, Hercules, CA, USA). GLY was determined in plasma collected in a vacuum tube (NaF-Na2 EDTA, Vacutainer, Becton Dickinson, USA). The remaining biomarkers were measured in serum (SST II PET tube with clot activator, silica, and separating gel, Vacutainer, Becton Dickinson, USA). Biomarkers were quantified using the automated Advia Chemistry XPT system (Siemens Healthcare Diagnostic, Erlangen, Germany) with calibrators and controls provided by the manufacturer. The LDL-C was calculated with parameters in mg/dL using the Martin–Hopkins equation [19]. LDL-C = TC − HDL-C − (TGs/variable factor), where the “factor” value is estimated in relation to the concentration of TGs vs. non-HDL cholesterol (non-HDL). LDL-C calculations were performed using an Excel spreadsheet obtained from the Johns Hopkins website (https://ldlcalculator.com/; accessed on 2 February 2025). The main stages of the work are shown in Figure 2.

### 2.3. Data Transformation

The raw data, comprising over 1,000,000 records, were initially obtained in text format, separated by semicolons. They were subsequently subjected to Knowledge Discovery Database steps as outlined by Fayyad (2001) and Shu and Ye (2023) [20,21]. The data were organized by removing redundancies, homonyms, and incomplete or noisy information to harmonize the text. It is important to note that, with the removal of incomplete data, each patient record includes all the parameters studied, thereby enhancing the computational power of the algorithms and statistics employed. Following this, incompatible and inconsistent data were removed, and data from different files were combined. After this cleaning phase, the database was reduced to approximately 450,000 records. Subsequently, inclusion and exclusion criteria were applied, resulting in a final consolidated sample of 3024 records. The sample selection process employed is important as it enables the analyses to be replicated in other studies, although it may also introduce some bias with respect to the raw data.

### 2.4. K-Means Clustering

The sample under analysis is complex, with a large size, no incompleteness, no extreme outliers, and multiple variables. Under these conditions, k-means, an unsupervised machine learning algorithm, has been employed and has demonstrated excellent performance in generating clusters based on similarity [22,23,24]. Clustering with k-means was performed using the R software package (v. 4.2.2) and the Jamovi software (v. 2.7.15) with the “snowCluster” module. The optimal number of clusters was determined using the Elbow curve, represented by the sum of squared errors and the gap statistic k [25,26]. Further details regarding sample processing are provided in the Supplementary Material. With the consolidated records, the collected biomarkers and derived variables are listed in Table 1.

### 2.5. Statistical Analysis

The Kolmogorov–Smirnov test was used to verify the normality of continuous variables. None of the parameters followed a normal distribution (*p* < 0.05); therefore, they were presented as median and interquartile range (25–75%). Differences between continuous variables were assessed using the Mann–Whitney U test. Discrete variables were presented as numbers and percentages, and comparisons were made using the Chi-square test. A 95% confidence interval (95% CI) was calculated using the one-proportion test. Spearman’s correlation was employed in the analysis.

The analyses were conducted using the R programming language (v. 4.2.2; https://r-project.org/); Statistica (v. 14.0), Data Science Workbench, MedCalc Statistical Software version (v. 23.4.5), and Jamovi software (v. 2.7.15). Relative risk (RR) was calculated using MedCalc as outlined by Newland (2024) [37]. A probability <5% (*p* < 0.05) was considered significant in all analyses.

## 3. Results

### 3.1. K-Means Clustering Analysis

**Figure 3:**
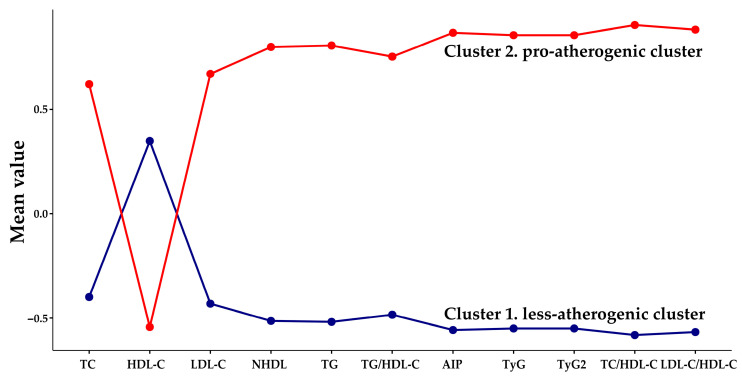
K-means cluster profile. The variables are those described in Table 1. Abbreviations: TC (total cholesterol); HDL-C (HDL-cholesterol); LDL-C (LDL-cholesterol); TG (fasting triglycerides); NHDL (cholesterol non-HDL-C); TG/HDL-C (ratio triglycerides to HDL-C); AIP (Atherogenic Index of Plasma, Log_10_(TG/HDL-C); TyG and TyG2 (triglycerides to glucose index); TC/HDL-C (Total cholesterol/HDL-C ratio, Castelli I index) and LDL-C/HDL-C (LDL-cholesterol/HDL-C ratio, Castelli II index). Figure generated in Jamovi software (v. 2.7).

The number of clusters in the k-means analysis of the selected records (n = 3024) was determined by the Elbow curve and the gap statistic k (Supplementary, Figure S1A,B). The analysis software indicated that the optimal number of clusters for the sample under study is two. The cluster profile plot of the parameters used in the k-means analysis is shown in Figure 3.

Table 2 presents the anthropometric and laboratory marker data for the two groups identified by the k-means analysis. Given the stronger association with pro-atherogenic lipid-pattern phenotypes (Table 1), cluster 1 (n = 1911) was designated as less-atherogenic (L-AC), while cluster 2 (n = 1113) was pro-atherogenic (P-AC).

The total sample had a median age of 62 (53–69) years, with a predominance of females (62%). The groups generated by k-means clustering (P-AC and L-AC) were similar in age and sex, as well as in Hb_A1c_ concentrations (Table 2). The cluster where biomarker concentrations were pro-atherogenic was operationally designated as “increase-risk phenotype,” whereas the cluster with more favorable profiles was designated as “less-risk or normal-risk phenotype.” These labels refer exclusively to biomarker-defined cardiometabolic profiles and do not represent confirmed cardiovascular disease or prediction of clinical outcomes, as no longitudinal or imaging data were available. All other evaluated parameters differed significantly (*p* < 0.001). The P-AC exhibited pro-atherogenic changes in all tested parameters compared to the L-AC cluster.

### 3.2. ROC Curve

**Figure 4:**
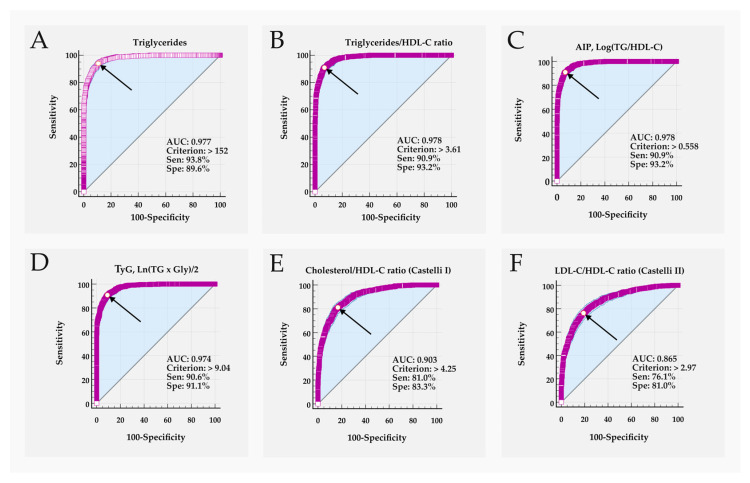
ROC curve for biomarkers associated with cardiovascular pro-atherogenic phenotype. AUC, area under the curve; Criterion, cut-off value with all parameters in mg/dL; Sen, sensibility (%); Spe, specificity (%). The arrow indicates the criterion point (cut-off). The dashed line on the curve indicates the 95%CI. (**A**) Triglyceride (TG); (**B**) Triglyceride/HDL-C ratio; (**C**) AIP, log_10_ (TG/HDL-C); (**D**) TyG, Ln [fasting triglycerides (mg/dL) × fasting glycemia (mg/dL)/2]; (**E**) Total cholesterol/HDL-C ratio (Castelli index I), and (**F**) LDL-C/HDL-c ratio (Castelli index II).

The ability of biomarkers to discriminate between the two k-means-generated groups was assessed using ROC curve analysis. Biomarkers that distinguished between groups with a sensitivity or specificity of ≥80% were considered relevant. Table 3 and Figure 4 show the ROC curve parameters for biomarkers with performance > 80%. The Supplementary Material provides the ROC curve results for all studied parameters, with additional results for the remaining parameters presented in Supplementary Material (Table S1).

Figure 4 shows the ROC curve profile for the best discriminating parameters, highlighting their characteristics comparatively.

The TyG and TyG2 ratios yield identical results (Table 3). Additionally, the lipid profile biomarkers TC, HDL-C, LDL-C, and non-HDL have a lesser contribution to group discrimination.

The TG/HDL-C and atherogenic index of plasma (AIP) ratios displayed a very similar ability to discriminate between P-AC and L-AC. We opted to use the AIP as a parameter for these evaluations because AIP demonstrates linearity when compared to the curvilinear response of the others, which potentially enhances biomarker performance [38]. Based on these observations, we focused on evaluating a rational set of the best-performing biomarkers, which includes TG, AIP, TyG, TC/HDL-C, and LDL-C/HDL-C. The correlations between these variables are presented in Table 4.

All variables identified as effective predictors in distinguishing phenotypes among prediabetics, classified using k-means (Table 3), exhibited moderate (r_S_, 0.4–0.69), strong (r_S_, 0.7–0.89) or very strong (r_S_, 0.9–1.0) correlations among the biomarkers (Table 4) [39]. This correlation pattern is sustained in the less-atherogenic cluster (Supplementary Table S2B) but diminishes in the pro-atherogenic cluster, where correlations are low or absent in the Castelli indices I and II (Supplementary Table S2A).

### 3.3. Binomial Logistic Regression

The binomial logistic regression (BLR) was employed to predict the probability of an increased of pro-atherogenic phenotypes among pre-diabetics selected by k-means. The combination of k-means with BLR is also interesting because it minimizes overfitting, the inclusion of spurious predictors in the model, which produces imprecision. Initially, the variables AIP, TyG, TC/HDL-C, and LDL-C/HDL-C were used, with the dependent variable being P-AC and L-AC generated by k-means. Results are available in the Supplementary (Table S3a–e). Although this approach achieved an accuracy rate above 98% for both groups, it showed markers such as a variance inflation factor (VIF) exceeding 10 (Supplementary Table S3c), indicating significant multicollinearity, which compromises the model’s reliability.

Subsequently, we evaluated the BLR using only the AIP and LDL-C/HDL-C variables. In this model, a VIF of ~1.0 indicated no significant correlation between predictor variables, characterizing the model as stable (Supplementary Table S4a–e). This was the sole combination of variables where multicollinearity was eliminated or minimized in the BLR. The resulting equation (Supplementary Table S4a) was:

$$ln\left(\frac{P}{1-P}\right)\;=\;\beta_{0}\;+\;\beta_{1}x_{1}\;+\;\beta_{2}x_{2}\;+\cdots +\;\beta_{k}x_{k}\;(\mathrm{general}\;\mathrm{equation})$$

y = 17.96 + (−24.85) × **AIP** + (−1.27) × **LDL-C/HLD-C** (resulting equation)

where y characterized the *P* as the probability of the event occurring and ln(*P*/1 − *P*) represents the odd of the event [40].

**Figure 5:**
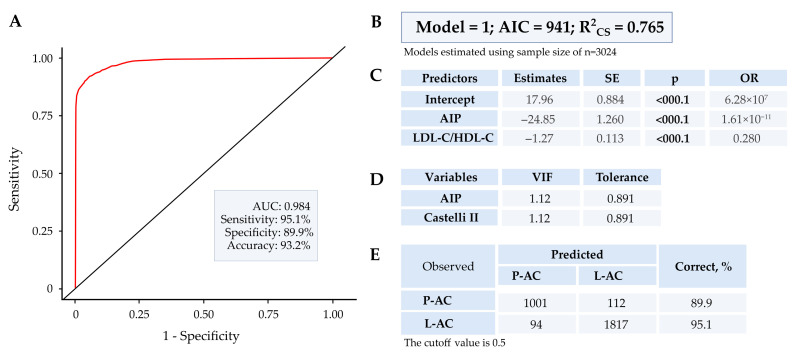
Binomial logistic regression with AIP and Castelli II variables in prediabetics separated by k-means. P-AC, pro-atherogenic cluster; L-AC, less-atherogenic cluster; AIP, log_10_ (TG/HDL-C); LDL-C/HDL-C ratio (Castelli II index). (**A**) ROC curve with parameters; (**B**) model; (**C**) predictors (model equation); (**D**) VIP and tolerance, indicators of multicollinearity and (**E**) performance of the model.

Figure 5 summarizes the discriminatory characteristics of BLR with the AIP and LDL-C/HDL-C variables.

The accuracy rate in classifying groups using BLR was 89.9% for the pro-atherogenic cluster and over 95% for the less-atherogenic cluster. The BLR’s performance in this model suggests excellent discriminatory ability and low multicollinearity.

The comparison between lipid biomarkers for classifying cardiovascular pro-atherogenic phenotypes in the prediabetic group is listed in Table 5.

Biomarkers, including fasting TG levels, demonstrated enhanced ability to differentiate between groups, notably AIP. The BLR model incorporating AIP and LDL-C/HDL-C effectively characterized relative risk between pro-atherogenic and less-atherogenic clusters.

## 4. Discussion

Prediabetes, an increasingly prevalent condition globally, is defined by glycemic biomarker concentrations that fall between those indicative of diabetes and normal levels (Figure 1). The concept of prediabetes represents a heterogeneous metabolic state, encompassing various groups or phenotypes [41]. For example, Lin et al. (2025) applied the discriminative dimensionality reduction tree algorithm with clinically available biomarkers and identified four distinct phenotypes, labeled as “relatively normal,” “risk for T2D,” “chronic kidney disease,” and “cardiovascular disease” [42].

Stefan et al. (2016) suggested incorporating cardiometabolic risk assessment in prediabetic patients, considering the complexity of the various phenotypes encompassed by this designation [43].

The characterization of prediabetes in this study involved selecting individuals with concomitant GLY concentrations (100–125 mg/dL) and HbA1c (5.7–6.4%), cut-off values recommended by the American Diabetes Association [1]. The choice of this criterion minimizes the inclusion of individuals with T2D exhibiting good glycemic control within the sample. Additionally, it has been proposed as a more sensitive and specific screening tool for early-stage prediabetes [44,45,46].

The k-means algorithm, a straightforward unsupervised machine learning tool accommodating diverse data types, has proven effective in clustering complex databases [47]. In our study, the k-means clustering process identified two optimized clusters (Figure 3), as evaluated by the elbow curve (Supplementary Figure S1A,B). The characteristics of these two clusters, based on the biomarkers under study, allowed us to define a group with a stronger association with pro-atherogenic biomarkers, designated the pro-atherogenic cluster (P-AC), as opposed to the less-atherogenic cluster (L-AC) (Table 2). The P-AC and L-AC were also matched for sex and age (Table 2), factors that did not affect classification or improve the models studied. The predominance of women (62%) and a median age of 62 years reflect the characteristics of the source population for the records. The laboratory parameters associated with the lipid profile were significantly different (*p* < 0.001) between the groups, with the P-AC exhibiting pro-atherogenic concentrations. The parameters TyG and TyG2 (TG and glucose index), which differ slightly in their generating equations (Table 1), were indistinguishable in the evaluations conducted. The use of both TyG formulas leads to multicollinearity in regression analyses, which is why TyG2, the less frequent equation, was not used. Therefore, we present the results only for TyG in this paper.

To assess the performance of the biomarkers in differentiating the two clusters generated by k-means, we plotted a ROC curve (Table 3, Figure 4, and Supplementary Table S1).

The variables that included triglycerides (TGs, TG/HDL-C, AIP, TyG) demonstrated excellent group discrimination (AUC > 0.97), with sensitivity and specificity equal to or greater than 90% (Table 3). The TC/HDL-C (Castelli I) and LDL-C/HDL-C (Castelli II) ratios were good discriminators (AUC > 0.86) with sensitivity and specificity close to 80%.

Interestingly, the cut-off values for the best discriminating biomarkers (Table 3) are similar to the criteria widely used in established guidelines and studies on CVD risk (Table 1). A notable example is TG concentration, where 152 mg/dL (associated criterion or cut-off) differentiated the groups, aligning with global guidelines that recommend fasting TG > 150 mg/dL as indicative of increased risk for CVD [48]. Therefore, our results reinforce the relevance of the cut-off values used in the main guidelines for triglycerides and suggest that these can be applied to discriminate a cardiovascular pro-atherogenic cluster in prediabetes.

The biomarkers TG, TG/HDL-C, and AIP showed similar performance. We chose to solely use the AIP for subsequent analysis, representing this group. AIP correlates with HDL and LDL particle size and the risk of CVD in multiple studies [31]. An increase in AIP is linked to a higher risk of coronary artery disease, with greater severity and worse prognosis [32]. Jiang et al. (2024) reported that an AIP of >0.806 (or >0.29 when parameters are in mmol/L) was associated with the risk of prediabetes and T2D in an adult Chinese population (OR 2.24; 95%CI, 1.67–3.0), demonstrating its association with these conditions [49]. In fact, AIP has been suggested as a predictor of cardiovascular events in prediabetics with unstable angina [50]. In our study, AIP > 0.558 (>0.198 with parameters in mmol/L) appears to be an excellent discriminator of P-AC and L-AC clusters generated by k-means, with sensitivity and specificity exceeding 90% (Table 3 and Supplementary Tables S3d and S4d).

The TyG index has also been identified as a biomarker with broad applications in various pathological processes [51]. For instance, insulin resistance, characteristic of T2D, has been suggested as strongly associated with the TyG index [52]. Chen et al. (2023) showed that a TyG index < 8.88 was associated with a reversion to normoglycemia from prediabetes in a Chinese population [53]. Our findings showed that TyG > 9.04 (>1.67 when parameters are in mmol/L) was associated with the pro-atherogenic cluster, demonstrating high sensitivity and specificity (Table 3). The similarity of the TyG cut-off values between our study and that of Chen et al. (2023), indicating risk and benefit, respectively, in a prediabetic population, highlights the complexity of this group [53].

The Castelli I and II indices, which refer to the ratios TC/HDL-C and LDL-C/HDL-C, respectively, were proposed over 40 years ago as indicators of CVD risk [34,54]. Qiu et al. (2025) [55] demonstrated an association between the Castelli I and II indices and the risk of prediabetes in Chinese individuals, albeit they also reported that the performance of these indices was modest, with an AUC of 0.55–0.68. In our study, the Castelli indices facilitated the classification of P-AC and L-AC clusters with improved performance (AUC > 0.86) and both sensitivity and specificity around 80% [55].

The other biomarkers evaluated (i.e., TC, HDL-C, LDL-C, non-HDL-C, FG, and HbA1c) did not offer good discriminatory capacity for the selected groups (Supplementary Table S1). The biomarkers under study showed a relevant and significant correlation (r_S_ > 0.56) with each other (Table 4). Biomarkers that include triglycerides (TG, AIP, and TyG) in their composition exhibited the highest correlation (r_S_ > 0.70), as expected (Supplementary Table S2A,B).

Next, we sought to evaluate the discriminatory capacity of the selected biomarkers by combining their effects through multiple regression on the generated groups. The most effective model was the BLR. The BLR performed excellently in characterizing the P-AC and L-AC clusters proposed by k-means when combining the variables AIP, TyG, TC/HDL-C, and LDL-C/HDL-C (Supplementary Table S3a–e). With this composition, the accuracy rate for the groups was over 98%. However, for this analysis, the BLR exhibited a high indication of multicollinearity, with the VIF exceeding 10 for various parameters (Supplementary Table S3c). High multicollinearity leads to unstable estimators, impacting the reliability of the model’s prediction [56].

The high correlation demonstrated between the variables (Table 4 and Supplementary Table S2A,B) supports the VIF response observed in the BLR. Alternatively, we found that the combination of AIP and LDL-C/HDL-C (Castelli II index) biomarkers in the BLR effectively distinguished between pro-atherogenic and less-atherogenic clusters with low multicollinearity (VIF ~1.0). With this composition, the BLR achieved a correct capture of the P-AC (89.9%) and L-AC (95%) clusters (Figure 5 and Supplementary Table S4a–e). The significant discrimination capacity of the BLR suggests its potential to generate predictive responses for identifying a cardiovascular pro-atherogenic phenotype in prediabetes.

It is important to emphasize that the classification of the clusters identified in the study by k-means, ROC curve, or binomial logistic regression represents pro-atherogenic phenotypes defined by lipid-biomarkers and have not been confirmed by independent cardiovascular risk or outcomes predictors.

## 5. Limitations

For instance, we did not include clinical information on patients, including BMI (Body Mass Index) data, objective markers of cardiovascular risk, such as imaging studies (e.g., echocardiograms, carotid and Doppler ultrasounds), or family history of prediabetes. Thus, further studies evaluating these variables will be necessary to consolidate the results of this study. As a counterpoint to the limitations, we point out the significant sample size of the study and the use of lipid profile biomarkers, widely recognized as indicators of pro-atherogenic processes. The use of unsupervised algorithms (e.g., k-means), whether associated or not with BLR, for characterizing subtypes or phenotypes in diabetes and prediabetes is well-documented in the literature [41,57,58].

The k-means algorithm used in data clustering is affected by the presence of similar or correlated variables, which can limit sample classification. It should be highlighted that the use of the unsupervised k-means algorithm, which promotes cluster identification and is associated with RLB that categorizes the data, is an exploratory tool and differs from other AI algorithms [59]. The results of our cross-sectional study describe prediabetic phenotypes stratified by serum lipid profile patterns rather than validated cardiovascular risk prediction.

Nevertheless, the consistency of the two identified clusters (P-AC and L-AC) was confirmed using ROC curve analysis and binomial logistic regression, with variables that increased collinearity removed [60].

Identifying different phenotypes in the intermediate hyperglycemia stage, such as pre-diabetes and cardiometabolic risk, is crucial for directing appropriate, effective, and individualized therapy for these groups [43]. Our study demonstrated the characterization of a phenotype with alterations in widely recognized pro-atherogenic cardiovascular biomarkers, which are low-cost and accessible to all institutions. Cut-off values for these biomarkers were identified to characterize this pro-atherogenic lipid-pattern phenotype, offering simplified, feasible, and directly accessible stratification for clinicians and their patients. This approach can be particularly beneficial for public health services, allowing for planned and optimized intervention in identifying prediabetics with a pro-atherogenic phenotype, thus providing broad benefits in economic terms and for those affected.

Additionally, binomial linear regression analysis presents an equation that can be used to identify pro-atherogenic phenotype in prediabetics. Thus, our findings should be validated by other populations.

The proposed method for identifying the pro-atherogenic phenotype for CVD in this study is simple, does not require new or complex technologies, and can be implemented in laboratory routines without significant costs.

## 6. Conclusions

The k-means algorithm successfully identified a pro-atherogenic cluster within a sample of prediabetic individuals based on routine lipid profile biomarker concentrations. The application of binomial logistic regression to the groups generated by the k-means algorithm, utilizing laboratory biomarkers such as triglycerides, HDL-C, and LDL-C, offered excellent discrimination of the pro-atherogenic cluster.

## Figures and Tables

**Table 1:** Biomarkers, calculated indices, and expected cut-off values for cardiovascular risk.

Biomarkers	Characterization	Cut-Off Risk CVD	References
**Lipid profile**			
TC	Total cholesterol (mg/dL)	≥200	[27]
HDL-C	HDL-cholesterol (mg/dL	≤40	[28]
*LDL-C	LDL-C = Total cholesterol (mg/dL) − HDL-Cholesterol (mg/dL) − (Triglycerides (mg/dL)/factor)	≥160	[19,29]
TG	Triglycerides (mg/dL). fasting	≥150	[30]
**Cardiovascular risk indices or ratios**			
AIP	Log_10_ [Triglycerides (mg/dL)/HDL-Cholesterol (mg/dL)] Log_10_ [Triglycerides (mmol/L)/HDL-Cholesterol (mmol/L)]	>0.57 >0.21	[31,32]
TG/HDL-C	Triglycerides (mg/dL)/HDL-cholesterol (mg/dL) ratio	≥3.5	[33]
TC/HDL-C Castelli I index	Total cholesterol (mg/dL)/HDL-cholesterol (mg/dL) ratio	≥5.0	[34]
LDL-C/HDL-C Castelli II index	LDL-cholesterol (mg/dL)/HDL-cholesterol (mg/dL) ratio	≥3.5	[27,28,30,34]
Non-HDL-C	Total Cholesterol (mg/dL) − HDL-cholesterol (mg/dL)	≥130	[29]
TyG	Ln[(fasting triglycerides (mg/dL) × fasting glycemia (mg/dL)/2]	>8.31	[35]
TyG2	Ln[(fasting triglycerides (mg/dL) × fasting glycemia(mg/dL)]/2	>4.65	[36]

AIP, Atherogenic index of plasma; TC/HDL-C, also known as Castelli Index I; Cut-off, cut-off values for increased risk of cardiovascular disease; *LDL-C, estimated by the Martin–Hopkins equation; LDL-C/HDL-C, also known as Castelli Index II; Non-HDL-C, cholesterol non-HDL-c; TyG, the Naperian logarithm (Ln) of triglycerides to glucose ratio presented in two equations, TyG and TyG2.

**Table 2:** Anthropometric and biomarker characteristics of groups classified by cluster k-means.

Variables	Cardiovascular Phenotypes			*p*-Value
	Total (n = 3024)	P-AC (n = 1113)	L-AC (n = 1911)	
Sex (M/F, %)	1148/1876 (38/62)	423/690	725/1186	0.971 *
Age (years)	62 (53–69)	62 (53–69)	62 (53–66)	0.512
Fasting glycemia (mg/dL)	107 (101–114	108 (102–116)	106 (100–113)	**<0.001**
HbA1c (%)	6.1 6.0–6.3	6.1 (6.0–6.3)	6.1 (6.0–6.3)	0.632
TC (mg/dL)	188 (161–218)	208 (180–238)	177 (155–203)	**<0.001**
HDL-C (mg/dL)	47 (39–57)	40 (35–46)	52 (44–61)	**<0.001**
LDL-C (mg/dL)	128 (101–154)	146 (121–174)	116 (94–140)	**<0.001**
Triglycerides (mg/dL)	138 (102–168)	213 (180–260)	113 (88–134)	**<0.001**
Non-HDL-C (mg/dL)	138 (112–168)	167 (141–194)	125 (102–147)	**<0.001**
TG/HDL-C	2.97 (1.9–4.5)	5.20 (4.2–6.9)	2.17 (1.54–2.87)	**<0.001**
TC/HDL-C ratio	3.9 (3.2–4.9)	5.1 (4.4–6.0)	3.4 (2.8–4.0)	**<0.001**
LDL-C/HDL-C ratio	2.7 (2.0–3.5)	3.6 (3.0–4.4)	2.3 (1.7–2.8)	**<0.001**
TyG	8.92 (8.6–9.2)	9.36 (9.17–9.57)	8.69 (8.44–8.88)	**<0.001**
TyG2	4.80 (4.6–5.0)	5.03 (4.9–5.1)	4.69 (4.5–4.8)	**<0.001**

Prediabetes criteria adopted by [19,20]: HbA1c, glycated hemoglobin fraction A1c 5.7–6.4 and fasting blood glycemia (mg/dL) 100–126. Values are median (interquartile range; 25–75%). P-AC, pro-atherogenic cluster and L-AC, less-atherogenic cluster. Abbreviations: TC (total cholesterol); HDL-C (HDL-cholesterol); LDL-C (LDL-cholesterol); TG (fasting triglycerides); Non-HDL-C (cholesterol non-HDL-C); TG/HDL-C (ratio triglycerides to HDL-C); AIP (Atherogenic Index of Plasma, Log_10_(TG/HDL-C); TyG and TyG2 (Triglycerides to glucose index); TC/HDL-C (Total cholesterol/HDL-C ratio, Castelli I index) and LDL-C/HDL-C (LDL-cholesterol/HDL-C ratio, Castelli II index). *p*-value, Mann–Whitney U test. or * Chi-square test. Marked in bold—significative (*p* < 0.05).

**Table 3:** Receiver operating characteristic curve parameters for studied biomarkers with high discrimination between groups.

Variable	TG	TG/HDL-C	AIP	TyG TyG2	TC/HDL-C	LDL-C/HDL-C
AUC	0.977	0.978	0.978	0.974	0.903	0.865
95% CI	0.97–0.98	0.97–0.98	0.97–0.98	0.96–0.98	0.89–0.91	0.85–0.88
*p*-value	<0.0001	<0.0001	<0.0001	<0.0001	<0.0001	<0.0001
Youden index J	0.834	0.842	0.842	0.816	0.643	0.571
Associated criterion						
mg/dL	>152	>3.61	>0.558	>9.04	>4.25	>2.97
mmol/L	>1.72	>1.57	>0.198	>1.67	>4.25	>2.97
Sensitivity (%)	93.8	90.9	90.9	90.6	81.0	76.1
Specificity (%)	89.6	93.2	93.2	91.1	83.3	81.0

Parameters selected with sensibility or specificity ≥ 80%. Total sample size 3024 with two groups generated by k-means (pro-atherogenic cluster, P-AC = 1113 and less-atherogenic cluster, L-AC = 1911); AUC. area under the curve; Associated criterion, cut-off values; 95%CI, confidence interval of 95%. Abbreviations: AIP, atherogenic index of the plasma = Log_10_(triglycerides/HDL-C) ratio; LDL-C/HDL-C, LDL-cholesterol/HDL-C ratio (Castelli II index); TC/HDL-C, total cholesterol/HDL-C ratio (Castelli I index); TG, triglycerides; TG/HDL-C, Triglycerides/HDL-C ratio; TyG, Ln [fasting triglycerides (mg/dL) × fasting glycemia (mg/dL)/2] and TyG2 Ln[(fasting triglycerides (mg/dL) × fasting glycemia(mg/dL)]/2.

**Table 4:** Spearman’s correlation coefficient for selected variables.

Variables	Values of Spearman (r_S_ or ρ) Correlation Coefficient				
	TG	AIP	TyG	TC/HDL-C	LDL-C/HDL-C
AIP	0.920	1.000	1.000	0.776	0.686
TG	1.000	0.920	0.920	0.649	0.566
TyG	0.984	0.907	0.907	0.636	0.552
TC/HDL-C	0.649	0.776	0.776	1.000	0.975
LDL-C/HDL-C	0.566	0.686	0.686	0.975	1.000

All values are significant (*p* < 0.05). Abbreviations: AIP, Atherogenic index of the plasma; TG, triglycerides; TyG, TG and glycemia index; TC/HDL-C, Castelli I index; LDL-C/HDL-C, Castelli II index.

**Table 5:** Effects of lipid biomarkers on selected pro-atherogenic phenotypes in the studied groups separated by k-means.

	Cut-Off Values		Frequencies Above the Cut-Off Values		Relative Risk, %	
Biomarkers	mg/dL	mmol/L	P-AC	L-AC	RR (95%CI)	*p*-Value
AIP	>0.558	>0.198	1012 (90.9)	130 (6.8)	13.3 (11–16)	**<0.001**
TyG	>9.04	>1.67	1012 (90.9)	186 (9.7)	9.3 (8.1–10.7)	**<0.001**
Triglycerides (TG)	>152	>1.72	1044 (93.8)	199 (10.4)	9.0 (7.9–10.3)	**<0.001**
TC/HDL-C (Cas I)	>4.25	>4.25	902 (81.0)	319 (16.7)	4.8 (4.4–5.4)	**<0.001**
LDL-C/HDL-C (Cas II)	>2.97	>2.97	847 (76.1)	365 (19.1)	4.0 (3.6–4.4)	**<0.001**
AIP OR Cas II			1093 (98.2)	455 (23.8)	4.1 (3.8–4.5)	**<0.001**
ALL (OR): TG OR AIP OR TyG OR TC/HDL-C OR LDL-C/HDL-C			1113 (100)	645 (32.1)	3.0 (2.8–3.1)	**<0.001**
Binomial logistic regression BLR (AIP+LDL-C/HDL-C)			1001 (89.9)	94 (4.9)	18.3 (15–22)	**<0.001**

95% CI, 95% confidence interval. P-AC, pro-atherogenic cluster; L-AC, less-atherogenic cluster; AIP, Log_10_(TG/HDLC); ALL (OR), at least one positive = TG > 152 OR AIP > 0.558 OR TYG > 9.04 OR TCHDL-C > 4.25 OR LDL-C/HDL-C > 2.9; BLR, Binomial logistic regression; Cut-off values from ROC curves (Table 3); LDL-C/HDL-C (Castelli II index); RR, relative risk; TC/HDL-C (Castelli I index); TG, Triglycerides; TyG, Ln [fasting triglycerides (mg/dL) × fasting glycemia (mg/dL)/2]. Significative *p*-value (<0.05) are in bold.

## Data Availability

The authors confirm that the data supporting the findings of this study are available within the article.

## Supplementary Materials

The following supporting information can be downloaded at: https://www.mdpi.com/article/10.3390/biomedicines14030651/s1, Figure S1: Optimal number of clusters for k-means; Table S1: Receiver Operating Characteristic Curve Parameters for Studied Biomarkers with Low Discrimination Between Groups Characterized by K-Means; Table S2: Spearman coefficient correlation for selected variables separated by clusters P-AC and L-AC; Table S3: (a–e) Binomial logistic regression predicting cardiovascular pro-atherogenic phenotypes in prediabetes selected by k-means; Table S4: (a–e) Binomial logistic regression predicting pro-atherogenic cardiovascular phenotypes in prediabetes selected by k-means with two-variables. Details of data processing.

## Abbreviations

The following abbreviations are used in this manuscript:

AI	Artificial Intelligence
AIP	Atherogenic Index of Plasma
AUC	Area Under the Curve
BLR	Binomial Logistic Regression
BMI	Body Mass Index
CVD	Cardiovascular disease
EDTA	Ethylenediamine Tetraacetic Acid
GLY	Fasting glycemia
HbA1c	Glycated Hemoglobin Fraction A1c
HDL-C	High-Density Lipoprotein—Cholesterol
L-AC	Less-Atherogenic Cluster
LDL-C	Low-Density Lipoprotein—Cholesterol
P-AC	Pro-Atherogenic Cluster
ROC	Receiver Operating Characteristic
T2D	Type 2 Diabetes
TC	Total Cholesterol
TG	Triglycerides
TYG	TyG index—ln [triglicerídeos de jejum (mg/dL) × glicemia de jejum (mg/dL)/2]
VLDL-C	VLDL-cholesterol
