# Supplementary material for: Stratification of Pro-Atherogenic Phenotypes in Prediabetes Using Machine Learning
Source: Biomedicines. 2026 Mar 13;14(3):651. doi: 10.3390/biomedicines14030651 (PMC13024570; doi:10.3390/biomedicines14030651)
Supplement: Supplementary file 1 [file biomedicines-14-00651-s001.zip › biomedicines-4140133-supplementary.pdf]

biomedicines

Supplementary Material

K-Means Clustering Algorithm

The K-means algorithm, an unsupervised machine learning technique, was applied under the conditions outlined below, with biomarkers described in Table 1: **Sample** (n = 3,024) was selected based on prediabetes criteria; **Covariates**: **TC** (total cholesterol); **HDL-C** (HDL-cholesterol); **LDL-C** (LDL-cholesterol); **TG** (fasting triglycerides); **TG/HDL-C** (Triglycerides to HDL-C ratio); **AIP** (Atherogenic Index of Plasma, Log_{10}(TG/HDL-C)); **TyG** (Triglycerides to Glucose index); **TC/HDL-C** (Total cholesterol to HDL-C ratio, Castelli I index).

The number of clusters in the K-means analysis of the selected records (n = 3,024) was determined using the Elbow curve and the gap statistic method (Figures S1 A and B). The optimized number of clusters for the sample under study was indicated as two by the analysis software.

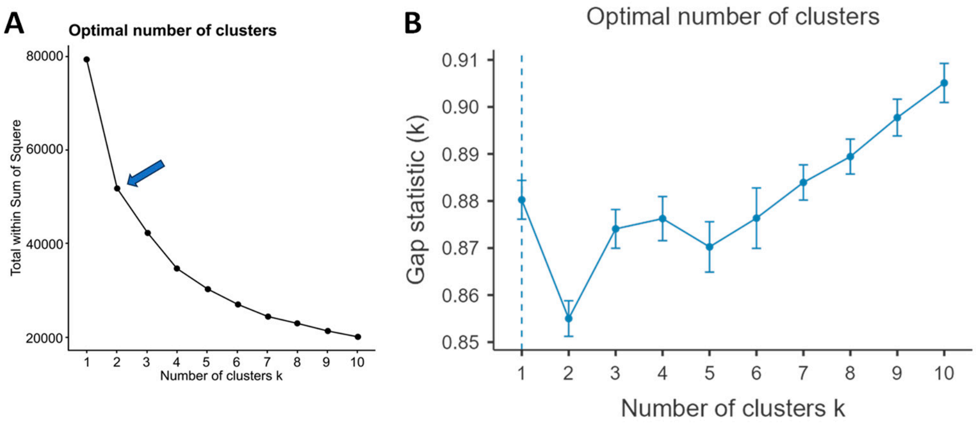

Figure S1. Optimal number of clusters for k-means. A) Elbow Curve for Estimating the Number of Clusters. The method incrementally increases the number of clusters, starting from one, analyzing and optimizing the results with each increment. A plateau in the curve indicates no significant benefit from further clustering. The arrow indicates two clusters, which is the number of groups recommended by the "R" software. B) Gap Statistic for Selecting the Optimal Number of Clusters. The optimal number of clusters is determined as the smallest value of k, which is two clusters in this case. Statistics were generated using Jamovi software (version 2.7).

The Gap Statistic compares how well the clusters formed from your actual data stand out against what would be expected if the data were randomly distributed. In other words, it assesses how tightly packed your real clusters are compared to clusters created from random data. This helps us understand if the patterns observed in our data are meaningful or merely due to chance.

 https://doi.org/10.3390/biomedicines14030651

ROC curve for parameters with lower performance

Table S1 is a supplement to Table 3 from the main paper, where studied biomarkers showed sensitivity or specificity of less than 80%.

Table S1. Receiver Operating Characteristic Curve Parameters for Studied Biomarkers with Low Discrimination Between Groups Characterized by K-Means.

Variables	TC	HDL-C	LDL-C	NHDL	GLY	HbA1c
Units	mg/dL	mg/dL	mg/dL	mg/dL	mg/dL	%
AUC	0.704	0.792	0.718	0.798	0.568	0.505
95%CI	0.68–0.72	0.77–0.81	0.70–0.73	0.78–0.81	0.55–0.59	0.49–0.52
p-value	<0.0001	<0.0001	<0.0001	<0.0001	<0.0001	<0.0001
Youden index J	0.307	0.438	0.380	0.454	0.108	0.027
Associated criterion	>196	≤43.5	>131.5	>147	>105	>6.1
Sensitivity, %	61.4	66.0	66.8	70.4	63.2	49.7
Specificity, %	69.9	77.8	67.2	75.0	47.7	53.1

Total sample size 3,024 with two groups generated by k-means (Pro-atherogenic cluster = 1,113 and Less-atherogenic cluster = 1,911); AUC. area under the curve; 95%CI. confidence interval of 95%; GLY, fasting glucose; HbA1c, glycated hemoglobin fraction A1c; HDL-C, HDL-cholesterol; LDL-C, LDL-cholesterol; NHDL, cholesterol non-HDL-C; TC, total cholesterol

Spearman correlation analysis

Variables with a good discriminatory capacity of groups by k-means are presented.

Table S2. Spearman coefficient correlation for selected variables separated by clusters P-AC and L-AC

S2A. Pro-atherogenic cluster

	Pro-atherogenic cluster - Spearman Rank Order Correlations				
Variable	AIP	TG	Tyg	TC/HDL-C	LDL-C/HDL-C
AIP	1.000	**0.758**	**0.728**	**0.477**	**0.285**
TG	**0.758**	1.000	**0.958**	**0.178**	0.020
TyG	**0.728**	**0.958**	1.000	**0.152**	-0.007
TC/HDL-C	**0.477**	**0.178**	**0.152**	1.000	**0.942**
LDL-C/HDL-C	**0.285**	0.020	-0.007	**0.942**	1.000

https://doi.org/10.3390/biomedicines14030651

S2B. Less-atherogenic cluster

	Less-atherogenic cluster - Spearman Rank Order Correlations				
**Variable**	**AIP**	**TG**	**Tyg**	**TC/HDL-C**	**LDL-C/HDL-C**
AIP	1.000	**0.815**	**0.789**	**0.610**	**0.525**
TG	**0.815**	1.000	**0.962**	**0.365**	**0.299**
TyG	**0.789**	**0.962**	1.000	**0.347**	**0.279**
TC/HDL-C	**0.610**	**0.365**	**0.347**	1.000	**0.974**
LDL-C/HDL-C	**0.525**	**0.299**	**0.279**	**0.974**	1.000

Total sample size 3024 with two groups generated by k-means (pro-atherogenic cluster = 1,113 and Less-atherogenic cluster = 1,911);. AIP. atherogenic index of the plasma = Log_{10}(Triglycerides/HDL-C) ratio; LDL-C/HDL-C. LDL-cholesterol/HDL-C ratio (Castelli II index). TC/HDL-C. total cholesterol/HDL-C ratio (Castelli I index); TG, triglycerides; TyG. Ln [fasting triglycerides (mg/dL) x fasting glycemia (mg/dL) / 2]; Correlation values (r_S) in bold show p-value <0.05.

Binomial logistic regression analysis

Binomial logistic regression performed with Jamovi software (version 2.7) with biomarkers of best discrimination.

Table S3. Binomial logistic regression predicting cardiovascular pro-atherogenic phenotypes in prediabetes selected by k-means
Dependent variable: Groups (code 1= less-atherogenic) and code 2=pro-atherogenic) classified by k-means.
Covariates: AIP, TyG, TC/HDL-C ratio and LDL-C/HDL-C ratio

S3a. BLR model coefficients

					OR 95%CI	
**Predictors**	**Estimates**	**SE**	**p**	**OR**	**Low**	**Upper**
Intercept	524.03	56.678	< 0.001	3.81e+227	2.17e+179	6.69e+275
AIP	-33.32	4.305	< 0.001	3.37e0-15	7.30e0-19	1.56e0-11
TyG	-3.01	0.987	0.002	0.04949	0.00715	0.3424
TC/HDL-C	-51.97	5.649	< 0.001	2.69e0-23	4.18e0-28	1.73e0-18
LDL-C/HDL-C	-6.87	1.174	< 0.001	0.00104	1.04e00-4	0.0104

Estimates represent the Log Odds of means clusters Pro-atherogenic cluster and Less-atherogenic cluster. SE, standard error; p, probability for Z-test; OR, Odds Ratio; 95%CI, confidence interval of 95%; AIP. atherogenic index of the plasma = Log_{10}(Triglycerides/HDL-C) ratio; LDL-C/HDL-C, LDL-cholesterol/HDL-C ratio (Castelli II index); TC/HDL-C. total cholesterol/HDL-C ratio (Castelli I index); TyG. Ln [fasting triglycerides (mg/dL) x fasting glycemia (mg/dL) / 2].

https://doi.org/10.3390/biomedicines14030651

Binomial Logistic Regression equation for AIP, TyG, TC/HDL-C and LDL-C/HDL-C

$$\ln\left(\frac{P}{1-P}\right) = \beta_0 + \beta_1 x_1 + \beta_2 x_2 + \cdots + \beta_k x_k$$

y=524.03 + (-33.32) x **AIP** + (-3.01) x **TyG** + (-51.97) x **TC/HDL-C** + (-6.87) x **LDL-C/HDL-C**

where "y" characterized the "P" the probability of the event occurring and ln(P/1−P) represents the "odds" of the event.

S3b. Model Fit Measures

Model	Deviance	AIC	R^2_{CS}
1	169	179	0.957

Models estimated using sample size of n=3,024

AIC, Akaike Information Criterion; R^2_{CS}, pseudo R^2 Cox and Snell

S3c. Assumption Check - Collinearity Statistics

Variables	VIF	Tolerance
AIP	3.07	0.3256
TyG	**18.39**	**0.0544**
TC/HDL-C	**17.67**	**0.0566**
LDL-C/HDL-C	**21.08**	**0.0474**

VIF, Variance Inflation Factor, Tolerance, 1/VIF

Significant multicollinearity values marked in bold

Criteria: VIF (Variance Inflation Factor) greater than 5 or 10 which corresponds to tolerance less than 0.2 or 0.1, often suggests multicollinearity.

VIF = 1: There is no correlation between the predictor variable and the other variables in the model.

VIF < 5: The correlation is moderate, but generally not considered problematic.

VIF > 5: Indicates a high correlation, which may be a cause for concern depending on the research context.

VIF >10: Suggests significant multicollinearity, which should be corrected for to ensure model reliability

S3d. ROC curve for binomial logistic regression

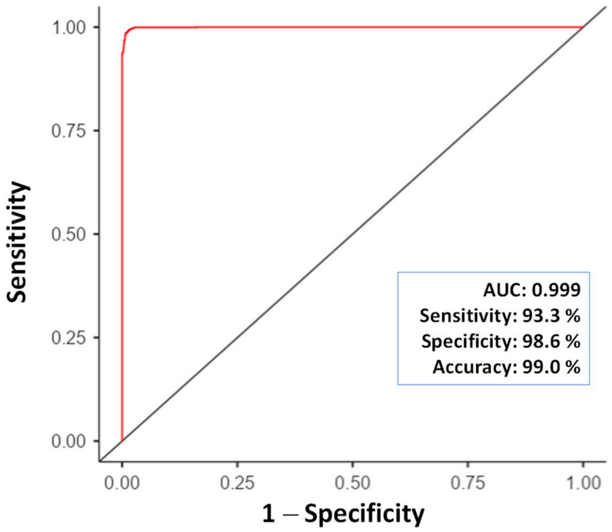

AUC. area under the curve

S3e. Classification table for binomial logistic regression

Observed	Expected		% Correct
	P-AC	L-AC	
P-AC	1,097	16	98.6
L-AC	14	1,897	99.3

Note: The cutoff value is 0.5

P-AC, pro-atherogenic cluster and L-AC, less-atherogenic cluster

Table S4. Binomial logistic regression predicting pro-atherogenic cardiovascular phenotypes in prediabetes selected by k-means with two-variables.

Dependent variable: code 1= less-atherogenic and code 2=pro-atherogenic classified by k-means.

Covariates: AIP and LDL-C/HDL-C ratio (Castelli II index)

S4a. BLR model coefficients

Predictors	Estimates	SE	p	OR	OR 95%CI	
					Low	Upper
Intercept	17.96	0.884	< 0.001	6.28e+7	1.11e+7	3.55e+8
AIP	-24.85	1.260	< 0.001	1.61e-11	1.36e-12	1.90e-10
LDL-C/HDL-C	-1.27	0.113	< 0.001	0.280	0.225	0.349

Estimates represent the Log Odds of "groups = less-atherogenic (L-AC)" vs. " Pro-atherogenic (P-AC)"; SE, standard error; p, probability for Z-test; OR, Odds Ratio 95%CI, confidence interval of 95%. AIP. atherogenic index of the plasma = Log_{10}(Triglycerides/HDL-C) ratio; LDL-C/HDL-C, LDL-cholesterol/HDL-C ratio (Castelli II index).

The resulting equation was

$$\ln\left(\frac{P}{1-P}\right) = \beta_0 + \beta_1 x_1 + \beta_2 x_2 + \cdots + \beta_k x_k$$

y = 17.96 + (-24.85) x **AIP** + (-1.27) x **LDL-C/HLD-C**

S4b. Model coefficients

Model	Deviance	AIC	R^2_{CS}
1	935	941	0.765

Models estimated using sample size of n=3024

AIC, Akaike Information Criterion; R^2_{CS}, pseudo R^2 Cox and Snell

S4c. Assumption Check - Collinearity Statistics

Criteria: VIF (Variance Inflation Factor) greater than 5 or 10 which corresponds to tolerance less than 0.2 or 0.1, often suggests multicollinearity. See S3c for extended criteria.

Variables	VIF	Tolerance
AIP	1.12	0.891
Castelli II	1.12	0.891

VIF, Variance Inflation Factor, Tolerance, 1/VIF

Significant values marked in bold

S4d. ROC curve for binomial logistic regression

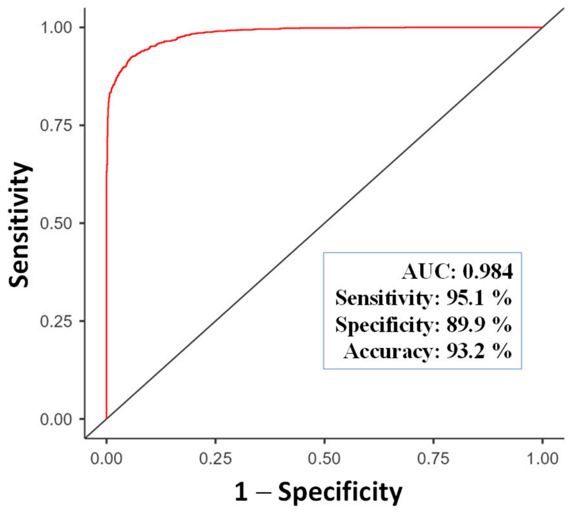

AUC. area under the curve

S4e. Classification table for binomial logistic regression

Observed	Predicted		Correct, %
	P-AC	L-AC	
P-AC	1,001	112	89.9
L-AC	94	1,817	95.1

Note: The cutoff value is 0.5

P-AC, pro-atherogenic cluster and L-AC, less-atherogenic cluster

Details of data processing

Next, we describe relevant elements associated with sample processing, complementing the information in Materials and Methods.

Data Transformation

Data transformation consisted of creating spreadsheets using the Python programming language (v.3.14.2; https://www.python.org/) and the PANDAS library (v.2.3.3; https://pandas.pydata.org/).

The raw data (>1,000,000 records) were obtained in text format, separated by semicolons. They were then subjected to the Knowledge Discovery Database (KDD) steps [21;59].

Preprocessing

The data was organized by removing redundant data, homonyms, incompleteness, and noise to harmonize the texts. Subsequently, the records were cleaned, a process that removes incompatible and inconsistent data and combines data from different files. After this phase, the database was reduced to approximately 450,000 records.

In this stage, the open-source tools SED (https://www.gnu.org/software/sed/) and CAT (https://www.gnu.org/software/coreutils/manual/html_node/cat-invocation.html#cat-invocation) were used, as well as regular expressions (https://www.gnu.org/software/grep/manual/html_node/Regular-Expressions.html) and the AWK language (https://www.gnu.org/software/gawk/manual/gawk.html). All the elements mentioned are native to Operating Systems originating from UNIX.

Then, the inclusion and exclusion criteria were applied, resulting in a final consolidated sample of 3,024 records.

Data Mining

The data mining step, the main one in the KDD (Knowledge Discovery in Databases) process, was performed with the R programming language (https://www.r-project.org/), version 4.2.2, using the following packages: dplyr, cluster, factoextra, pROC, pacman, gapminder, MultivariateAnalysis, corrplot, FactoMineR, clipr, ggplot2, rstatix, reportROC, and data.table.

K-means clustering

The sample under analysis is complex, with a large sample size, no incompleteness or extreme outliers and multiple variables. Under these conditions, k-means, an unsupervised machine learning algorithm, has been employed and demonstrated excellent performance in generating clusters based on similarity [22-24].

Clustering with k-means was performed with the "R" software package mentioned above, and with the Jamovi software (version 2.7) using the "snowCluster" module.

https://doi.org/10.3390/biomedicines14030651

The optimal number of clusters was estimated by the Elbow curve, expressed by the sum of squared errors (Datacamp. 2025; Available CRAN Packages. 2025F) and by the gap statistic k [26].

Post-processing

Post-processing consisted of creating tables, figures, and graphs to better visualize the results. ROC Curve (Receiver Operating Characteristics Curve) was constructed using the R programming language (pROC package) [60], MedCalc Statistical Software or Jamovi (version 2.7).

https://doi.org/10.3390/biomedicines14030651
